# Heme-Oxygenase-1 Is Decreased in Circulating Monocytes and Is Associated With Impaired Phagocytosis and ROS Production in Lupus Nephritis

**DOI:** 10.3389/fimmu.2019.02868

**Published:** 2019-12-13

**Authors:** Loreto Cuitino, Javiera Obreque, Patricia Gajardo-Meneses, Alejandra Villarroel, Natalia Crisóstomo, Ignacio F. San Francisco, Rodrigo A. Valenzuela, Gonzalo P. Méndez, Carolina Llanos

**Affiliations:** ^1^Departamento de Inmunología Clínica y Reumatología, Escuela de Medicina, Pontificia Universidad Católica de Chile, Santiago, Chile; ^2^Departamento de Anatomía Patológica, Escuela de Medicina, Pontificia Universidad Católica de Chile, Santiago, Chile; ^3^Departamento de Urología, Escuela de Medicina, Pontificia Universidad Católica de Chile, Santiago, Chile; ^4^Departamento de Ciencias Químicas y Biológicas, Facultad de Salud, Universidad Bernardo O'Higgins, Santiago, Chile

**Keywords:** lupus nephritis, monocytes, heme oxygenase-1 (HO-1), phagocytosis, reactive oxygen species (ROS), renal biopsies

## Abstract

Lupus nephritis (LN) is one of the most serious manifestations of systemic lupus erythematosus (SLE). Based on studies showing the potential role of heme oxygenase-1 (HO-1), an enzyme that catalyzes the degradation of heme and has anti-inflammatory properties in SLE development, we decided to explore HO-1 in LN. Accordingly, we evaluated HO-1 levels and function in circulating and infiltrating monocytes and neutrophils of LN patients. HO-1 levels were assessed in peripheral monocytes of LN patients and controls by flow cytometry and immunofluorescence microscopy. Phagocytosis and the production of reactive oxygen species (ROS) were evaluated to determine the effect of HO-1 in monocyte function. In addition, renal biopsies with proliferative LN were used to identify HO-1 in infiltrating cells and renal tissue by immunofluorescence and immunohistochemistry. Biopsies of healthy controls (HC) and patients who underwent nephrectomy were included as controls. Circulating pro-inflammatory monocytes and activated neutrophils were increased in LN patients. HO-1 levels were decreased in all subsets of monocytes and in activated neutrophils. LN monocytes showed increased phagocytosis and higher production of ROS than those of HC. When HO-1 was induced, phagocytosis and ROS levels became similar to those of HC. HO-1 was mostly expressed in renal tubular epithelial cells (RTEC). Renal tissue of LN patients showed lower levels of HO-1 than HC, whereas infiltrating immune cells of LN showed lower levels of HO-1 than biopsies of patients who had renal surgery. HO-1 is decreased in circulating monocytes and activated neutrophils of LN patients. HO-1 levels modulate the phagocytosis of LN monocytes and ROS production. HO-1 expression in RTEC might be an attempt of self-protection from inflammation.

## Introduction

Systemic lupus erythematosus (SLE) is a chronic autoimmune disease of unknown etiology that predominantly affects women in childbearing age and is associated with higher rates of mortality than the general population. Although in SLE, inflammation may affect multiple organs, lupus nephritis (LN) remains the focus of attention of clinical and basic scientists given that kidney involvement is a major predictor of morbidity and mortality (1, 2). Indeed, despite efforts to improve clinical outcomes, remission is achieved in only 50–70% of LN patients and 10–20% of them will progress to end-stage renal disease (ESRD) within 5 years of diagnosis. Therefore, the better understanding of the underlying mechanisms of kidney involvement would be a progress in the field that can potentially lead to the development of new therapeutic targets.

Heme oxygenase-1 (HO-1) is an enzyme that catalyzes the degradation of heme and has anti-inflammatory properties. We have previously evaluated HO-1 expression in circulating monocytes of SLE patients, finding that this enzyme is decreased in affected individuals (3) and in an animal model for SLE (4). HO-1 can be induced in human endothelial cells after the release of strong immunogenic cytokines, such as tumor necrosis factor (TNF)-α and interleukin (IL)-1α (5–7) and is required for the regulation of the immune response, with subsequent inhibition of pro-inflammatory signals and recovery of homeostasis (8, 9). Interestingly, when MRL-Fas^*lpr*^ lupus mice are treated with carbon monoxide (CO)—a bystander product and inducer of HO-1 expression—they present lower levels of circulating anti-dsDNA and anti-histone antibodies than untreated mice. In addition, kidneys from CO-treated lupus mice have less number of activated B220^+^CD4^−^CD8^−^ T cells than control animals and present delayed renal failure (4). Although it is well-known that, in LN, renal damage is initiated by glomerular deposition of immune complexes (IC) and subsequent complement activation, it has been acknowledged that innate immune cells also play an active role in the propagation of tissue damage. Recent data show that LN patients poorly degrade neutrophil extracellular traps (NETs), which promote the presentation of self-antigens (10). Even more, elegant experiments using a humanized lupus mouse model show that SLE human serum is capable of inducing LN in a process mediated by infiltrating neutrophils recruited by an FcγRIIA-dependent mechanism (11).

On the other hand, monocytes have been less studied than neutrophils but are well-known to participate in SLE pathogenesis. Indeed, expanding data have demonstrated that infiltrating monocytes and macrophages are phenotypically altered in SLE and are associated with both murine and human LN (12–15). In humans, monocytes have been divided into three subtypes based on relative surface expression of lipopolysaccharide (LPS) coreceptor CD14 and FCγIII receptor CD16, “classical” monocytes, “intermediate” monocytes, and “pro-inflammatory” monocytes. Several reports suggest that the primary function of majority of circulating monocytes is phagocytosis (16, 17) of cellular debris, pathogens, and other external agents in a way that does not involve the release of inflammatory mediators; however, it has been observed that monocytes of SLE patients are inefficient in the clearance of apoptotic cells, which is also associated with the production of pro-inflammatory cytokines such as IL-6, TNF-α, IL-10, transforming growth factor (TGF)-β, and interferon (IFN)-α (18–20).

Given our findings in circulating human monocytes and the interesting results obtained after treating lupus mice with CO, we decided to explore whether HO-1 levels of circulating and infiltrating monocytes play a role in the pathogenesis of human LN, with a special focus on the renal interstitium because it has recently been considered an important predictor of renal outcomes in LN patients (21). In addition, we also evaluated HO-1 levels in circulating neutrophils.

## Methods

### Patients

This study was approved by the Research Ethics Committee (REC) of Pontificia Universidad Católica de Chile (PUC). All subjects signed PUC-REC-approved consent forms. A total of 15 subjects who met the American College of Rheumatology SLE 1997 criteria and had a renal biopsy showing type III, IV, and/or V LN as per the ISN/RPS classification were included to do peripheral blood and purified monocyte analyses; data of patients are shown in Table 1 (LN-1–LN-15). Fifteen healthy controls (HC) were recruited to analyze peripheral blood samples; demographic data are summarized in Table 2.

**Table 1:** Clinical and demographic characteristics of patients with lupus nephritis (LN) who donated blood samples.

	**Age range (years)**	**Treatment**	**Arth**.	**Imm**.	**NS**	**Kid**.	**Haem**	**Ser**.	**MC**	**ANA**	**Hypoc**	**Anti-DNA (UI/ml)**	**Hgb (g/dl)**	**Hct (%)**	**Biopsies class**.	**Duration of disease (months)**
LN-04	51–55	AZT, PDN 5 mg	+	+	–	+	+	+	–	+	–	97.02	14.2	44.1	IV-G + V (A/C)	432
LN-05	31–35	HCQ, MMF, PDN 2.5 mg	+	+	–	+	–	–	–	+	+	381.3	12.7	37.7	IV-S (A)	177
LN-06	46–50	HCQ, MMF, PDN 2.5 mg	+	+	–	+	+	–	+	+	–	24.96	14.2	43.8	IV	288
LN-07	26–30	HCQ, PDN 10 mg	+	+	–	+	+	+	+	+	–	212.94	10	30.9	IV-G (A/C)	80
LN-08	26–30	HCQ, MMF	–	+	–	+	–	–	+	+	–	64.74	13	40.5	IV-S (A)	60
LN-09	26–30	HCQ, MMF	+	+	–	+	–	–	+	+	+	14.83			III+V	116
LN-10	36–40	HCQ, MMF, PDN 5 mg	+	+	–	+	+	–	+	+	–	388.95	12.7	39.7	IV-S (A)	40
LN-11	36–40	HCQ, MMF, PDN 7.5 mg	+	+	–	+	+	–	+	+	+	240.91	9.2	29.8	IV-G	192
LN-01	31–35	HCQ, MMF, PDN 5 mg	-	+	–	+	–	–	-	+	+	81.1	12.1	34.4	IV-G (A)	60
LN-02	46–50	–	–	+	–	+	–	–	+	+	+	74.59	13	38.1	IV-S (A/C)	408
LN-03	41–45	HCQ, PDN 2 mg	–	+	–	+	–	–	+	+	+	65.02	14.8	43.2	III + V (A/C)	120
LN-12	41–45	HCQ, MMF, PDN 15 mg	–	+	–	+	–	–	–	+	+	139.42	12.7	38.9	IV-S + V (A/C)	274
LN-13	18–20	HCQ, MMF	–	+	–	+	+	–	+	+	–	17.77	16.8	48.4	IV-G + V (A)	36
LN-14	51–55	HCQ, MMF, PDN 15 mg	+	+	–	+	–	–	+	+	–	<12.3	13	39.7	III-C + V	196
LN-15	56–60	HCQ, PDN 2 mg	–	+	–	+	–	+	–	+	–	143.79	13.3	40.3	IV-G + V (A)	288
LN-16	36–40	PDN 10 mg	+	–	–	+	–	–	+	+	–	24.64			IV-G (A)	24
LN-17	31–35	HCQ	–	+	–	+	–	–	–	+	+	65.51	13.3	36.5	III (A/C)	35
LN-18	31–35	HCQ, MMF	+	+	–	+	–	+	–	+	–	58.78	9.9	31	IV-S (A/C)	6
LN-19	31–35	HCQ, MMF, PDN 20 mg	+	+	–	+	+	–	+	+	–	162.06	11.1	36.2	III + V	60
LN-20	36–40	HCQ, MMF, PDN 5 mg	+	+	+	+	+	–	+	+	+	109.99	10.3	32.6	V + III A/C	84
LN-21	36–40	HCQ	+	+	–	+	+	–	–	+	–	<12.3	12.7	39.1	III (A)	2
LN-22	31–35	HCQ	+	+	–	+	+	–	+	+	+	<12.3	12.7	37.7	IV-S (A)	12
LN-23	31–35	HCQ, AZT, PDN 5 mg	–	+	–	+	–	–	+	+	+	809.66	13.6	39.1	IV-G (A/C)	2
LN-24	21–25	HCQ, MMF	+	+	–	+	–	–	+	+	–	210.9	13.7	41	V	36
LN-25	46–50	HCQ, MMF, PDN 5 mg	+	+	–	+	–	–	–	+	–	126.18	13.3	41.6	III (A/C)	156
LN-26	36–40	HCQ, MMF, PDN 5 mg	+	+	–	+	+	–	+	+	+	<12.3	11.4	34.9	IV-S (A)	23
LN-27	26–30	HCQ, MMF, PDN 5 mg	+	+	–	+	+	–	+	+	+	134.28	12.0	36.7	IV-S (A/C)	1
LN-28	31–35	HCQ, PDN 10mg	+	+	–	+	–	–	+	+	–	>1000	8.2	25.5	III + V (A)	16

*Art, Arthritis; Imm, Immune; presence of anti-DNA, anti-Sm, or anti-cardiolipin antibodies; NS, nervous system compromise (seizures or psychosis); Kid, Kidney; renal compromise; Haem, hematological compromise; Ser, Serositis; MC, mucocutaneous; ANA, Anti-nuclear antibodies; HCQ, hydroxychloroquine; PDN, prednisone (doses included); MMF, mycophenolate mofetil; AZT, azathioprine; anti-DNA, anti-double strand DNA (positive >75 UI/ml, Indeterminate 30–75 UI/ml, negative < 30 UI/ml); Hgb, hemoglobin; Hct, Hematocrit; Biopsy class, LN type III, IV, and/or V as per the ISN/RPS classification*.

**Table 2:** Demographic details of lupus nephritis (LN) patients and healthy controls recruited to obtain blood samples.

	**LN patients**	**Healthy control**
Number	15	15
Gender (% Female)	86.7	86.7
Age (years) ±SD	39.7 ± 11.4	38.11 ± 11.22
Arthritis (%)	53.3	N/A
Immune (%)	100	N/A
NS (%)	0	N/A
Kidney (%)	100	N/A
Haem. (%)	40.0	N/A
Serositis (%)	20.0	N/A
MC (%)	72.72	N/A
ANA (%)	100	N/A
Hypocomplementemia (%)	46.7	N/A
Anti-dsDNA (%)	54.54	N/A

*Immune, presence of anti-DNA, anti-Sm, or anti-cardiolipin antibodies; NS, nervous system compromise (seizures or psychosis); Kidney, renal compromise; Haem., hematological compromise; MC, mucocutaneous; ANA, Anti-nuclear antibodies; N/A, not applicable*.

Twenty renal biopsies with type III, IV, and/or V LN ISN/RPS classification performed between January 2006 and January 2017 were used for immunofluorescence and immunohistochemistry studies (Table 1, LN-09–LN-28). A summary of histologic characteristics of renal biopsies is shown in Table 3. Biopsies of three patients who underwent surgery and nephrectomy had to be performed as part of the surgical protocol for different reasons, but their kidneys were healthy and were included as HC. Biopsies of eight patients without autoimmune disease who had renal cancer and underwent nephrectomy were included [cancer controls (CC)]. Only tissue that did not present tumor was used for the purpose of this study.

**Table 3:** Pathological characteristics of lupus nephritis (LN) renal biopsies.

	**LN patients**
Class III (number/%)	8 (40%)
Class IV (number/%)	11 (55%)
Class V (number/%)	1 (5%)
Mean activity index score ±SD	9.22 ± 4.5
Mean chronicity index score ±SD	1.17 ± 1.2

*Twenty renal biopsies with type III, IV, and/or V LN ISN/RPS classification, performed between January 2006 and January 2017 were used*.

### Direct Immunofluorescence Staining for Flow Cytometry [Fluorescence-Activated Cell Sorting (FACS)]

To identify circulating monocytes and neutrophils, samples obtained by peripheral venous puncture, and the total blood was separated in 150 μl. Red blood cells were lysed using ammonium-chloride-potassium lysing buffer (ACK; ThermoFisher Scientific, Waltham, MA, USA) for 5 min at room temperature (RT), then washed and centrifuged. The pellet was resuspended in PBS-2% fetal bovine serum, and cells were counted using TC20 (Biorad, Hercules, CA, USA). To analyze monocytes and neutrophils, flow cytometry exclusion gating strategy was used as previously described (16). Briefly, the staining cells were selected using forward and side scatter characteristics and deleting the doublets (FSC vs. SSC). Monocytes were identified using the following antibodies: CD14-PeCy7, CD16-APC, CD3-Pe, CD19-Pe, CD66b-PerCpCy5.5, CD56-APC-Cy7 (Biolegend, San Diego, CA, USA). Neutrophils were identified using the following antibodies: CD15-APC-Fire750, CD66b-PerCp, CD11b-PE, CD3-FITC, CD19-FITC, CD14-PeCy-7, HLA-DR-APC (Biolegend).

To obtain purified monocytes, peripheral blood mononuclear cells (PBMCs) were isolated by density gradient centrifugation using Ficoll-Paque (GE Healthcare, Chicago, IL, USA) as previously described (22). Then monocytes were purified using Pan Monocyte Isolation Kit, Human (Miltenyi Biotec, Bergisch Gladbach) according to manufacturer's instructions. To identify monocytes, the purified cells (5 × 10^5^ cells) were stained with the following fluorescent conjugated antibodies (Biolegend): CD14-PeCy7 and CD16-APC.

For intracellular detection of HO-1, cells were fixed with fixation/permeabilization solution kit (BD Biosciences, Franklin Lakes, NJ, USA) and stained with an unconjugated rabbit polyclonal antibody (Abcam, Cambridge, MA, USA). After 30 min, cells were incubated with a conjugated secondary antibody (Alexa Fluor 555 or 488; ThermoFisher Scientific). All samples were analyzed in FACS Canto II using FACSDiva software.

### Phagocytosis Assay

To determine the phagocytic function of monocytes, FluoSpheres™ Carboxylate-Modified Microspheres (ThermoFisher Scientific) were used (23). The FluoSpheres™ were incubated with 100 μg of purified immunoglobulin G (IgG) for 30 min at 37°C to opsonize the beads. IgGs were purified from serum of a patient with LN who has high titers of anti-DNA antibodies (Table 1, LN-23) or of an HC matched in sex and age with the patient. Purified monocytes from LN patients (Table 1, LN-1–LN-15) or from HC (Table 2) were adjusted to 5 × 10^5^ cells and were incubated with opsonized FluoSpheres™ (ratio of monocytes to beads = 1:5) for 1 h at 37°C to determine the phagocytic function or at 4°C to detect nonspecific binding. Monocytes were washed three times using 1 ml of complete PBS and analyzed in LSRFortessa X20 Flow Cytometer using FACSDiva software (BD Immunocytometry Systems, San Jose, CA, USA).

### Determination of Reactive Oxygen Species (ROS)

ROS production was determined in 5 × 10^5^ purified monocytes using CellROX™ Green Flow Cytometry Assay Kit (ThermoFisher Scientific) according to manufacturer's instructions. Briefly, monocytes were incubated with 250 μM of CellROX® reagent for 30 min at 37°C in the dark. To obtain a positive and a negative control for ROS production, monocytes were preincubated with tert-butyl hydroperoxide (TBHP, 250 μM) or N-acetylcysteine (NAC, 1 mM), respectively, for 1 h at 37°C before incubation with CellROX®. During the final 15 min of staining, SYTOX® Red Dead Cell stain solution was added (5 μM) in each sample and mixed gently. Samples were immediately analyzed by flow cytometry.

### Induction of HO-1 Using Cobalt Protoporphyrin (CoPP)

Before phagocytosis and/or ROS determination assays, purified monocytes (5 × 10^5^) were treated with 100 μM of CoPP (Frontier Scientific, Logan, UT, USA) for 2 h at 37°C to induce HO-1 expression. Alternatively, to inhibit HO-1 expression, monocytes were treated with 100 μM of tin protoporphyrin (SnPP; Frontier Scientific) using the same protocol as for CoPP treatment. After 2 h, purified monocytes were washed and used for different experiments. CoPP and SnPP were dissolved in 0.1 M NaOH to prepare a stock solution of 10 mM.

### Immunohistochemistry

Tissues were deparaffinized in xylene and rehydrated using ethanol. The activity of endogenous peroxidase was blocked. For immunohistochemistry staining, immunoperoxidase system (VECTASTAIN PK-6200; Vectors laboratories, Burlingame, CA, USA) was used. Samples were stained with a mouse anti-HO1 (Abcam, Cambridge, MA, USA) monoclonal antibody and analyzed using ImmPACT™ AEC (SK-4205). Finally, samples were counterstained with hematoxylin and blindly evaluated by a pathologist (GM, AV) who determined the H- Score, which includes intensity (0-negative, 1-weak, 2-moderate, and 3-strong) and the percentage of cells that were positive for HO-1 staining (24).

### Immunofluorescence in Renal Biopsies

Biopsies were deparaffinized, rehydrated, and stained overnight with conjugated antibodies to identify monocytes (CD14; Abcam), activated neutrophils (CD66b; Biolegend), and macrophages (CD68; ThermoFisher Scientific), together with an unconjugated mouse/rabbit anti-HO-1 monoclonal antibody (Abcam). A secondary conjugated antibody (anti-mouse Alexa Fluor 555 and anti-rabbit Alexa Fluor 488; ThermoFisher Scientific) was also used. For nuclei staining, samples were incubated with DAPI (ThermoFisher Scientific). Images were analyzed using ImageJ software (NIH, Bethesda, MD, USA) to calculate the corrected total cell fluorescence (CTCF) (25).

### RNA Extraction and qPCR

Total RNA was extracted from monocytes using TRIzol (ThermoFisher Scientific), and cDNAs were generated from 1 μg RNA using ImProm-II™ Reverse Transcription System (Promega, Fitchburg, WI, USA) according to the manufacturer's protocol. cDNA products were used as template for qPCR experiments performed in a StepOnePlus™ Real-Time PCR system (Applied Biosystems) using Fast SYBR® Green Master Mix (Applied Biosystems). Relative expression of HO-1 was calculated through the ΔΔCt method using glyceraldehyde 3-phosphate dehydrogenase (GAPDH) and 18S genes as reference. Amplification was carried out with 160 nM of the following primers (5′ → 3′): HO-1: forward (fw): CCCCAACGAAAAGCACATCC—reverse (rv): AGACAGCTGCCACATTAGGG; GAPDH: fw: GGTGGTCTCCTCTGACTTCAACA—rv: GTTGCTGTAGCCAAATTCGTTGT; 18S: fw GTA ACC CGT TGA ACC CCA TT—rv: CCA TCC AAT CGG TAG TAG CG. Efficiencies were 98.96% (HO-1), 101.8% (GAPDH), and 103.9% (18S). ΔΔCt was defined as (ΔCt GOI treatment – ΔCt GOI control) – (ΔCt reference treatment – ΔCt reference control) and was calculated automatically by StepOne Software v2.3 (Applied Biosystems).

### Statistical Analysis

The results of all groups were expressed as mean ± SD. Statistical analyses were performed with GraphPad Prism 5 software (La Jolla, CA, USA) or SPSS-17 statistical software (SPSS Inc., Chicago, IL, USA). Mann–Whitney non-parametric test was used to compare LN patients with control groups. For statistical analysis between treatments, paired one-way Friedman test was used with Bonferroni *post-hoc* test over the ranked data. *P* < 0.05 were considered significant.

## Results

### Pro-Inflammatory Monocytes and Activated Neutrophils of LN Patients Show Lower Expression of HO-1 Than Those of HC

**Figure 1:**
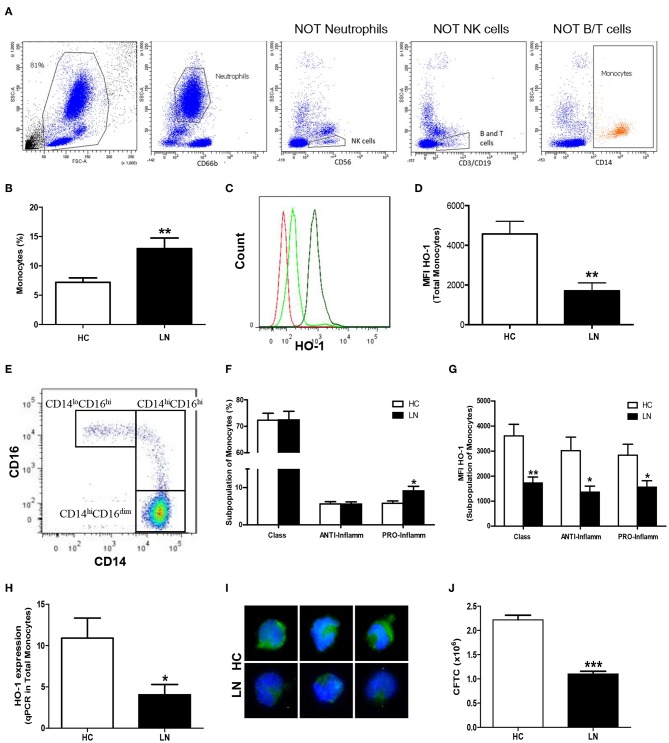
Monocytes from peripheral blood of lupus nephritis (LN) patients express lower levels of heme oxygenase (HO)-1 than healthy controls (HC). The proportion of cells and HO-1 expression [mean fluorescence intensity (MFI)] were determined in peripheral blood using flow cytometry. **(A)** Gating strategy for identification of monocytes showing successive exclusion of neutrophils, natural killer (NK) cells, and B and T cells. **(B)** Percentage of monocytes circulating. **(C)** Representative histograms for HO-1 expression in monocytes of LN patients and HC. Red: autofluorescence; light green: LN monocytes; green: HC monocytes. **(D)** MFI value of HO-1 expression. **(E)** Representative dot plot to identify the different subpopulations of monocytes. **(F)** Percentage of each subset's monocytes. **(G)** Representative MFI value of HO-1 expression in each subset of monocytes. **(H)** HO-1 expression was quantified in purified monocytes by real-time RT-PCR. **(I)** Immunofluorescence of HO-1 in purified monocytes. **(J)** Quantification of HO-1 expression [corrected total cell fluorescence (CTCF)] in purified monocytes. Graphs are showing the mean ± standard deviation. *n* = 15 LN patients (black bars) and *n* = 15 HC (white bars). *P < 0.05, **P < 0.01, ***P < 0.001 by Mann–Whitney test.

The expression of HO-1 in peripheral monocytes and neutrophils was evaluated in whole blood of LN patients (Table 1; patients LN-1–LN-15) and HC (Table 2; *n* = 15). To evaluate monocytes in peripheral blood, neutrophils and lymphocytes were excluded (Figure 1A). LN patients have higher percentage of monocytes than that of HC (Figure 1B). Interestingly, these LN monocytes had lower protein levels of HO-1 than those of HC monocytes (Figures 1C,D). Moreover, recent studies have revealed that peripheral monocytes can be divided based on the level of expression of type IIIa FCγ receptor (CD16) (17, 26), in classical (CD14^hi^CD16^lo^), anti-inflammatory (intermediate, CD14^hi^CD16^hi^), and pro-inflammatory monocytes (nonclassical, CD14^lo^CD16^hi^) (Figure 1E). Interestingly, a significant increase of proinflammatory monocytes was observed in LN patients when compared with HC; however, no significant differences were observed when classical and anti-inflammatory monocytes were evaluated in both groups (Figure 1F). HO-1 expression was decreased in all subsets of monocytes, including pro-inflammatory monocytes (Figure 1G). In addition, we analyzed HO-1 expression using MACS kit by real-time PCR (qPCR) (Figure 1H) and immunofluorescence (Figures 1I,J) in purified monocytes of LN patients and HC. We confirmed that HO-1 levels were decreased in LN monocytes compared with those of HC.

**Figure 2:**
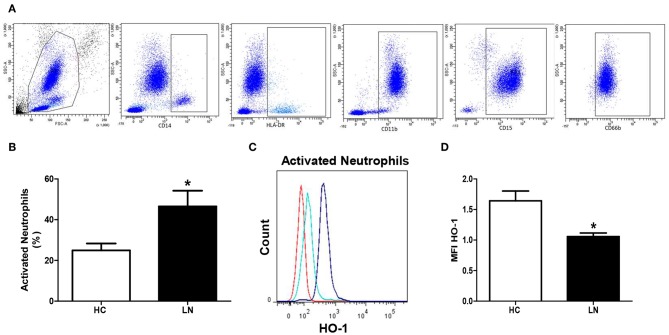
Activated neutrophils of lupus nephritis (LN) patients express lower levels of heme oxygenase (HO)-1 than healthy controls (HC). The proportion of cells and HO-1 expression [mean fluorescence intensity (MFI)] was determined in peripheral blood using flow cytometry. **(A)** Gating strategy to obtain neutrophils. CD14+ and HLA-DR+ cells were excluded, then CD11b+, CD15+, and CD66b were included. **(B)** Percentage of activated neutrophils. **(C)** Representative histograms for HO-1 expression in activated neutrophils of LN patients and HC. Red: autofluorescence; light blue: LN activated neutrophils; blue: HC activated neutrophils. **(D)** MFI value of HO-1 expression. Graphs are showing the mean ± standard deviation. *n* = 15 LN patients and *n* = 15 HC. *P < 0.05 by Mann–Whitney test.

To evaluate HO-1 expression in peripheral neutrophils, cells CD11b^+^/CD15^+^ were identified using exclusion strategy. After deletion of doublets cells from FSC vs. SSC plot, monocytes and lymphocytes were identified and excluded (Figure 2A). No significant differences between LN patients and HC were found in HO-1 expression in peripheral neutrophils (*p* = 0.5772). However, two different subsets of neutrophils based on the expression of CD66b have been described; thus, CD66b^+^ cells are activated neutrophils (27). Remarkably, LN patients showed higher percentage of circulating activated neutrophils than HC (Figure 2B), and when HO-1 expression was evaluated in this population, LN-activated neutrophils presented lower levels of this enzyme than HC (Figures 2C,D).

### Monocytes of LN Patients Have Different Phagocytic Capabilities Than Monocytes of HC

**Figure 3:**
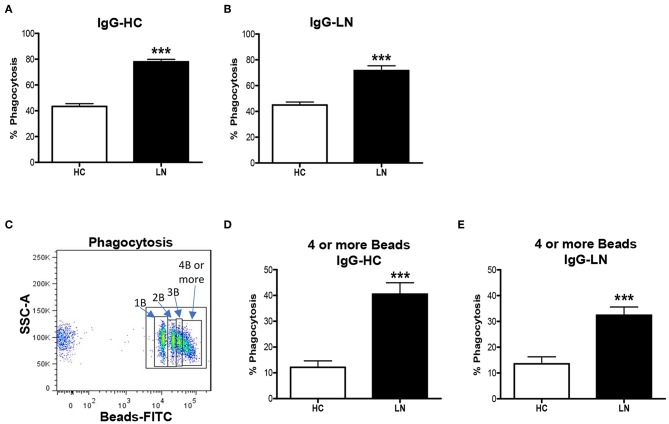
Monocytes of lupus nephritis (LN) patients show increased phagocytic activity compared with those of healthy controls (HC). **(A,B)** Phagocytic activity of purified monocytes incubated with opsonized beads using IgG from HC (IgG-HC) or LN patient (IgG-LN). **(C)** Representative dot plot to identify monocytes phagocyting different numbers of beads. **(D,E)** Percentage of monocytes that phagocyte four or more beads opsonized with IgG-HC or IgG-LN. Graphs are showing the mean ± standard deviation. *n* = 15 LN patients and *n* = 15 HC. ***P < 0.001 by Mann–Whitney test.

**Figure 4:**
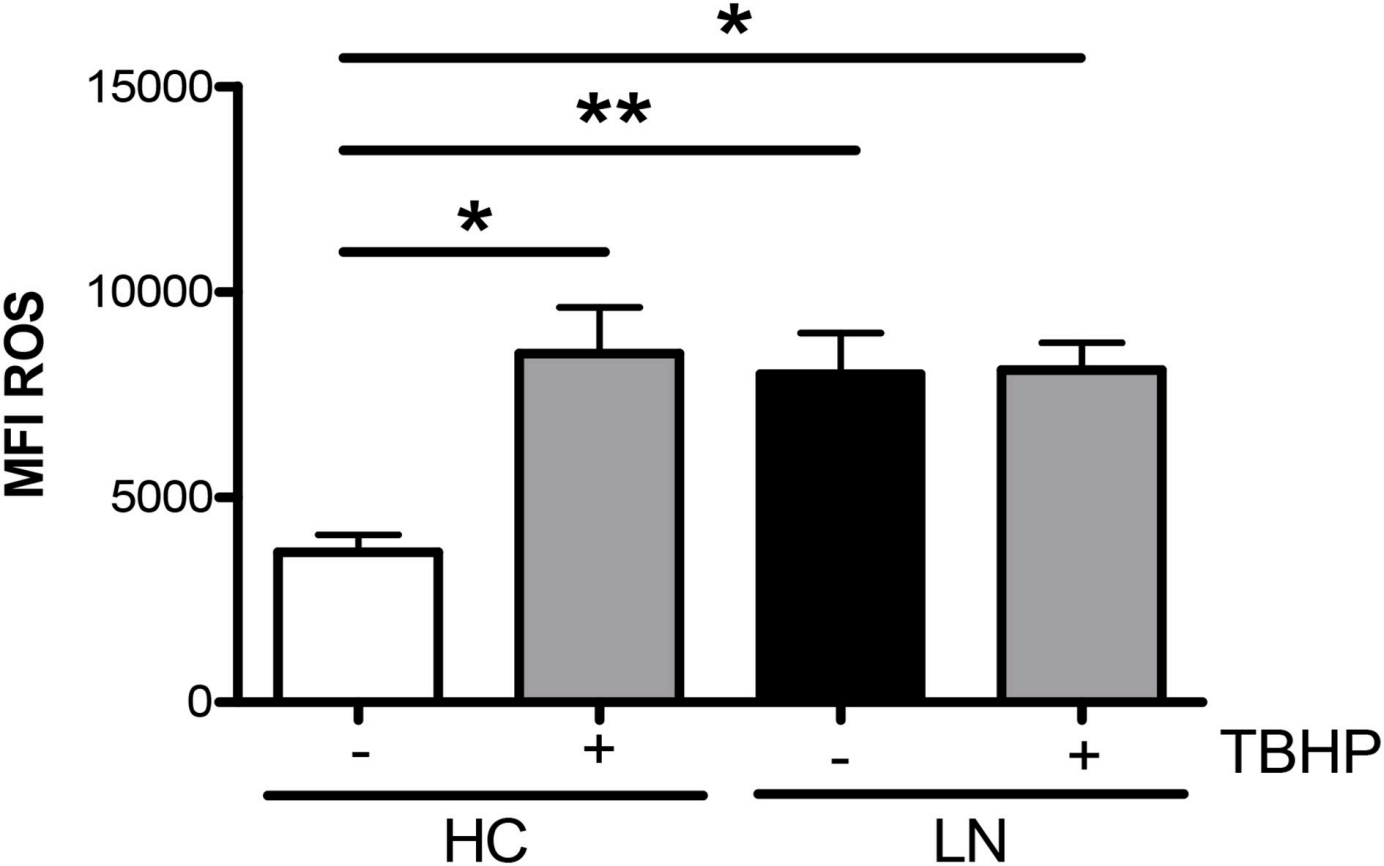
Reactive oxygen species (ROS) levels are elevated in monocytes of lupus nephritis (LN) patients. ROS mean intensity of fluorescence (MFI) levels were determined by fluorescence-activated cell sorting (FACS) by using CellROX®Green Reagent. TBHP is a ROS inducer and was used as a positive control. Graphs are showing the mean ± standard deviation. *n* = 15 LN patients and *n* = 15 healthy controls (HC). *P < 0.05 by Wilcoxon matched-pairs signed rank test (±TBHP). *P < 0.05, **P < 0.01 by Mann–Whitney test (between groups).

An important function of monocytes is the phagocytosis of antibody-coated microbes and cellular debris. As a way to evaluate this function, we investigated the ability of purified LN and HC monocytes to phagocytose IgG-opsonized beads. The phagocytic ability was measured by FACS, and the total phagocytosis was calculated as the percentage of cells with engulfed beads. We detected significant differences in the capacity of monocytes to engulf IgG-opsonized beads when comparing monocytes of LN patients with those of HC (Figures 3A,B). Surprisingly, the phagocytosis level was increased in monocytes of LN patients compared with HC, independently of whether the IgGs used to opsonize the beads came from an LN patient or a HC. Therefore, trying to further understand our unpredictable results, we turned our focus to the analysis of the percentage of monocytes that phagocyte different number of beads (Figure 3C). Interestingly, the most important difference was observed in the percentage of monocytes that phagocyte four or more beads, which is higher in LN monocytes regardless of the IgGs used to opsonize the beads (Figures 3D,E). Based on these results, we decided to evaluate ROS production as an additional approach to assess the effector function of monocytes, since it is widely known that ROS production is part of the mechanisms used by monocytes to remove microbes and other threats. Noteworthy, ROS levels at baseline were higher in monocytes of LN patients than in those of HC. Moreover, LN monocytes show at baseline similar levels of ROS to those achieved when HC monocytes were treated with TBHP, a potent ROS inducer (Figure 4).

### HO-1 Induction Improves the Phagocytic Activity and Decreases ROS Production of LN Monocytes

**Figure 5:**
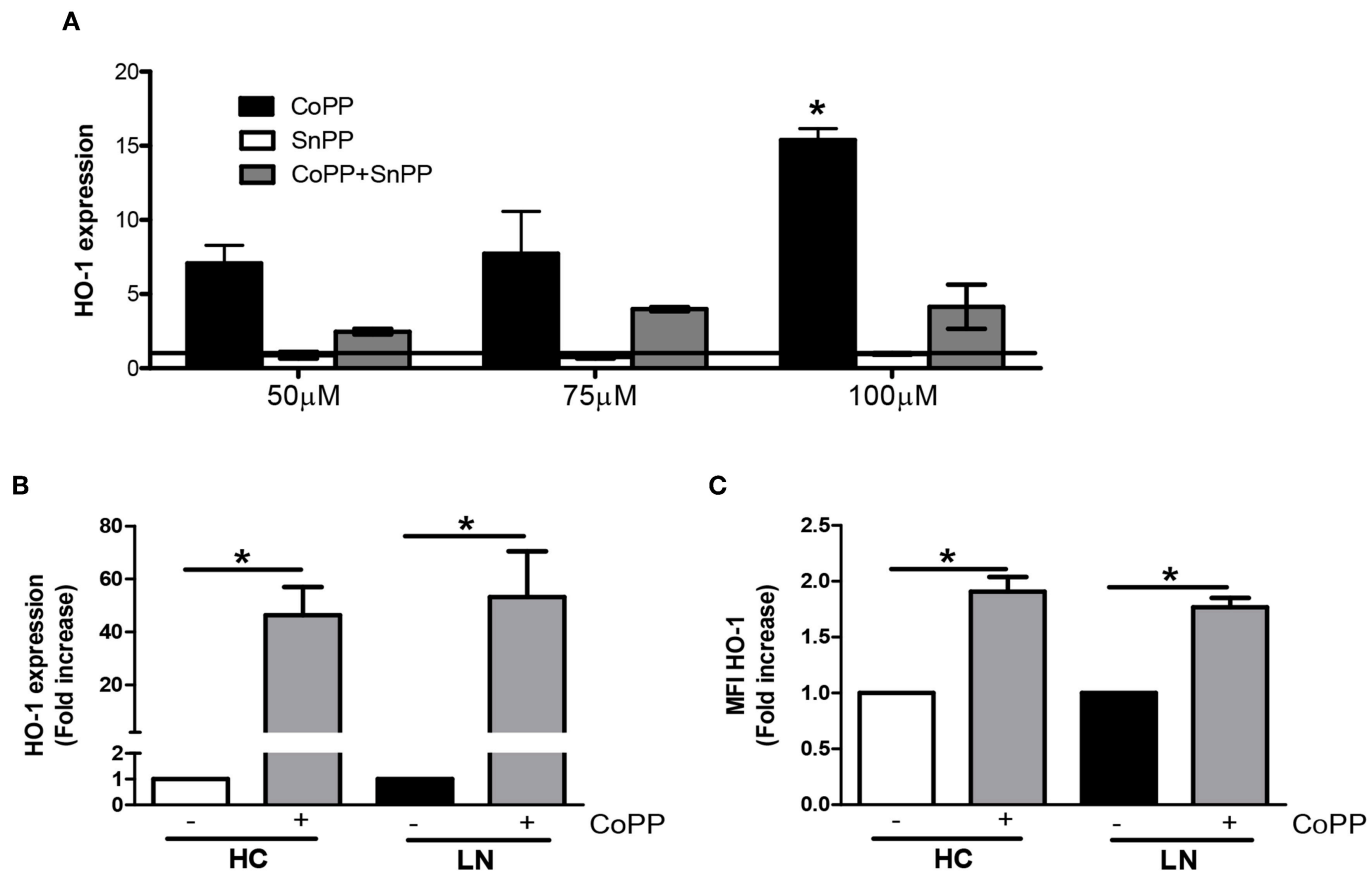
Cobalt protoporphyrin (CoPP) but not tin protoporphyrin (SnPP) induces heme oxygenase (HO)-1 expression in purified monocytes. **(A)** HO-1 expression was quantified in purified monocytes from lupus nephritis (LN) patients by real-time RT-PCR with different treatments. **(B,C)** Purified monocytes from healthy controls (HC) and LN patients were treated with 100 μM CoPP for 2 h at 37°C, and HO-1 expression was evaluated by real-time RT-PCR **(B)** and fluorescence-activated cell sorting (FACS) **(C)**. *P < 0.05; by Wilcoxon matched-pairs signed rank test (±CoPP).

**Figure 6:**
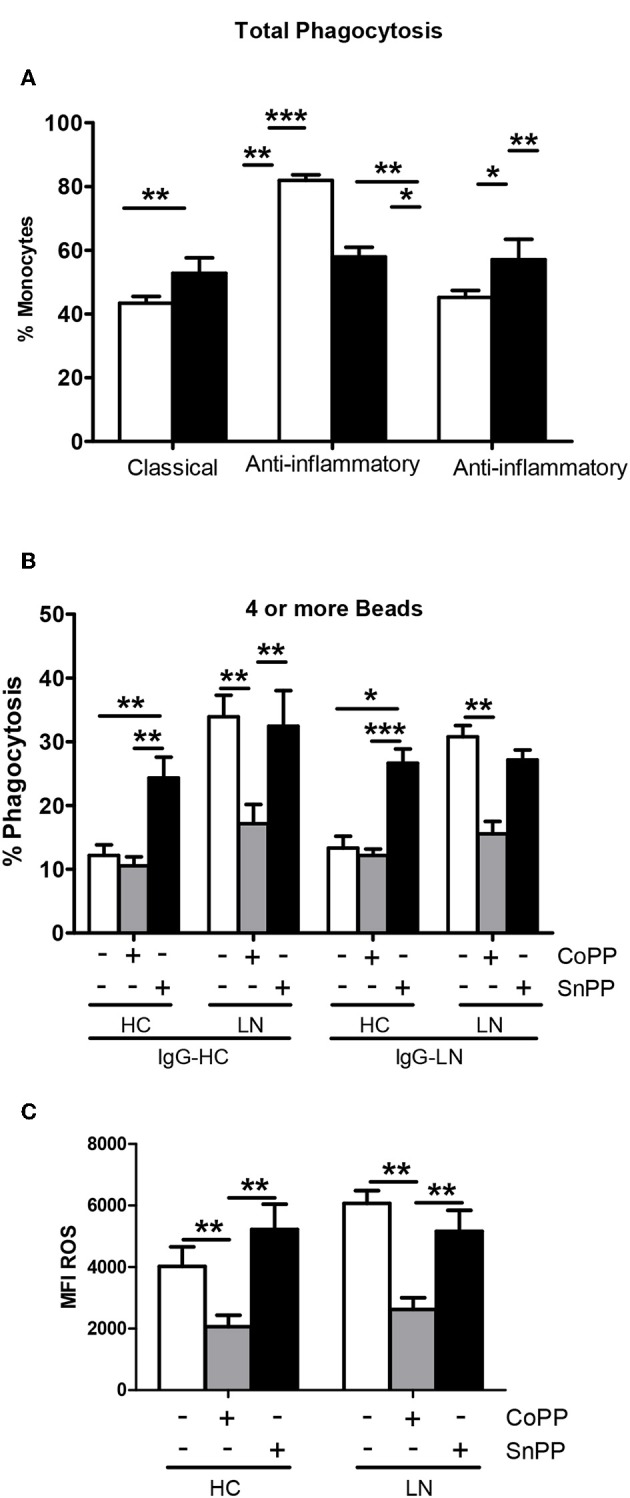
Heme oxygenase (HO)-1 induction in lupus nephritis (LN) and healthy control (HC) monocytes decreases phagocytic activity and reactive oxygen species (ROS) production. **(A)** Total phagocytic activity of purified monocytes of LN patients or HC previously treated with cobalt protoporphyrin (CoPP) or tin protoporphyrin (SnPP) to induce or inhibit HO-1 expression, respectively. **(B)** Percentage of monocytes treated with CoPP or SnPP that phagocyte four or more beads. **(C)** ROS levels in monocytes incubated with CoPP or SnPP. Graphs are showing the mean ± standard deviation. *n* = 15 LN patients and n = 15 HC. *P < 0.05, **P < 0.01, ***P < 0.001 by Friedman test (±CoPP ±SnPP).

To evaluate whether increasing the expression of HO-1 modulates ROS production, we treated purified monocytes of LN patients and HC with CoPP, which is known to promote the expression and activity of HO-1 (28, 29). In addition, to inhibit HO-1 induction, SnPP was used as a control (30). We first determined the appropriate concentration of CoPP that would achieve the maximum level of HO-1 induction without compromising cellular viability (Figure 5A). The expression of HO-1 was assessed by qPCR and flow cytometry. As shown in Figures 5B,C, purified monocytes upregulate the expression of HO-1 in response to CoPP. Interestingly, unlike the results previously shown, when monocytes were treated with CoPP, the percentage of LN monocytes that phagocyte IgG-beads decreased regardless of whether the IgG came from HC or LN patient, reaching similar values between LN patients and HC (Figure 6A). In addition, the phagocytic activity of HC monocytes did not change with CoPP treatment. Furthermore, with CoPP treatment, we did not observe differences in the percentage of monocytes that phagocyte four or more beads (Figure 6B), indicating that the induction of HO-1 regulates phagocytosis in monocytes of LN patients. On the other hand, to assess the function of LN monocytes in response to the induction of HO-1 in LN patients, we evaluated ROS production in cells treated with CoPP. Interestingly, HO-1 induction results in a decrease of ROS production in LN and HC (Figure 6C), leading the monocytes of LN patients and the monocytes of HC to produce similar levels of ROS. Noteworthy, these results, including the effect of CoPP in total phagocytosis, phagocytosis of four or more beads, and ROS production, were reversed when treating monocytes with SnPP, an inhibitor of HO-1 (Figure 6). Even in some cases, as in phagocytosis, HC monocytes treated with SnPP phagocytosed even more beads than untreated monocytes from HC (Figure 6).

### HO-1 Is Expressed in Renal Tubular Epithelial Cells and Infiltrating Innate Immune Cells of LN Patients

**Figure 7:**
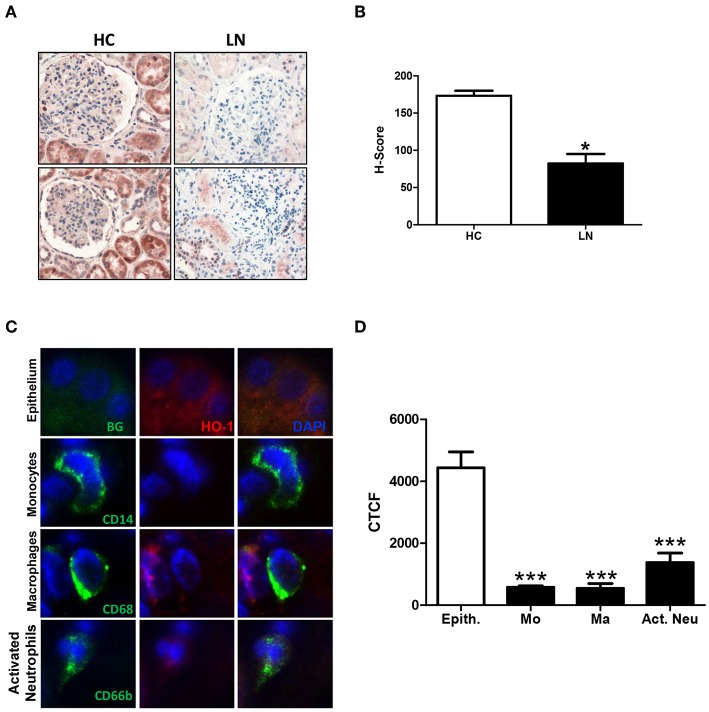
Heme oxygease (HO)-1 expression is lower in infiltrating immune cells than in renal tubular epithelial cells (RTEC) of lupus nephritis (LN) patients. Renal biopsies from LN patients and healthy controls (HC) were analyzed to quantify HO-1 expression. **(A)** Representative images of immunohistochemistry of LN and HC biopsies. **(B)** Quantification of H-score in LN and HC kidney biopsies. **(C)** Representative images of HO-1 expression in renal tubular epithelial cells (RTEC, epithelium) and infiltrating cells (monocytes, macrophages, and activated neutrophils) in LN biopsies detected by immunofluorescence (IF). **(D)** Quantification of HO-1 expression [corrected total cell fluorescence (CTCF)] in epithelium (Epith) and immune cells (Mo, monocytes; Ma, macrophages; Act. Neu, activated neutrophils). Graphs are showing the mean ± standard deviation. *n* = 20 LN biopsies and n = 3 HC. *P < 0.05, ***P < 0.001 by Mann–Whitney test **(A–C)**. ***P < 0.001 by Wilcoxon matched-pairs signed rank test **(D)**.

HO-1 expression was evaluated in renal biopsies of LN patients (Table 1; patients LN-09–LN-28; *n* = 20) and HC (Table 4; *n* = 3) using immunohistochemistry. Since obtaining renal tissue from HC is extremely difficult, we were able to evaluate HO-1 expression in only three biopsies of subjects who underwent surgery, and a nephrectomy had to be performed as part of the surgical protocol for different reasons, but their kidneys were healthy (HC). Interestingly, we observed that HO-1 was mostly expressed in the cytoplasm of renal tubular epithelial cells (RTEC), whereas other regions of the kidney such as the glomeruli showed limited expression (Figure 7A). Nevertheless, HO-1 expression was significantly higher in HC biopsies than in that in LN (173.33 ± 6.67 vs. 82.45 ± 12.70, *p* = 0.0313; Figure 7B). However, numbers are small, and although promising, further studies are needed to make definitive conclusions. Therefore, we decided to explore HO-1 expression in healthy tissue of biopsies of patients who had a nephrectomy due to the diagnosis of kidney cancer (CC) (see section Materials and Methods for characteristics and Table 4; *n* = 8). In this case, HO-1 levels in LN biopsies were lower than those in CC biopsies but did not reach statistical significance (122.5 ± 15.32 vs. 82.45 ± 12.7, *p* = 0.0658). In addition, we evaluated HO-1 expression in renal biopsies of five patients with antineutrophil cytoplasmic antibodies (ANCA)-associated vasculitis also by immunohistochemistry, and HO-1 expression was lower than that of HC biopsies (173.33 ± 6.67 vs. 43.70 ± 22.33, *p* = 0.0358) but did not reach statistical significance when compared to that of LN patients (data not shown).

**Table 4:** Demographic details of lupus nephritis (LN) patients, healthy controls, and cancer control recruited to obtain renal biopsies.

	**LN patients**	**Healthy control** ** (healthy kidney)**	**Cancer control** ** (tissue not infiltrated by tumor)**
Number	20	3	8
Gender (% Female)	90.0	75.0	37.5
Age (years) ±SD	37.4 ± 10.2	50.3 ± 27.3	56.6 ± 8.4
Arthritis (%)	60.0	N/A	N/A
Immune (%)	95.0	N/A	N/A
NS (%)	20.0	N/A	N/A
Kidney (%)	100	N/A	N/A
Haem. (%)	35.0	N/A	N/A
Serositis (%)	10.0	N/A	N/A
MC (%)	65.0	N/A	N/A
ANA (%)	100	N/A	N/A
Hypocomplementemia (%)	50.0	N/A	N/A
Anti-dsDNA (%)	50.0	N/A	N/A

*Immune, presence of anti-DNA, anti-Sm, or anti-cardiolipin antibodies; NS, nervous system compromise (seizures or psychosis); Kidney, renal compromise; Haem., hematological compromise; MC, mucocutaneous; ANA, Anti-nuclear antibodies; N/A, not applicable*.

**Figure 8:**
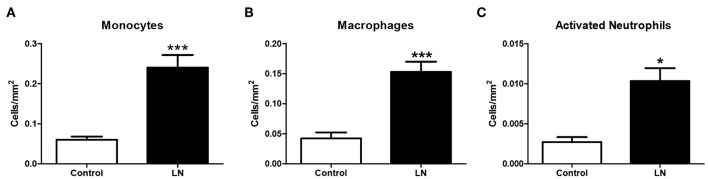
Lupus nephritis (LN) biopsies show higher number of infiltrating cells than cancer controls (CC). Renal biopsies from LN patients and CC were analyzed to identify infiltrating immune cells by immunofluorescence (IF). Infiltrating monocytes **(A)**, macrophages **(B)**, and activated neutrophils **(C)** were quantified and normalized per square millimeter. Graphs are showing the mean ± standard deviation. *n* = 20 LN biopsies and *n* = 8 CC. *P < 0.05, ***P < 0.001 by Mann–Whitney test.

However, when HO-1 was evaluated in infiltrating cells of LN biopsies using immunofluorescence, we found that monocytes, macrophages, and neutrophils showed a low expression of HO-1 compared with the strong expression seen in RTEC (Figures 7C,D). Monocytes, macrophages and neutrophils were identified using specific staining for CD14, CD68, and CD66b, respectively. In addition, infiltrating cells were quantified and normalized to the total area of the biopsy. As expected, the number of infiltrating monocytes, macrophages, and neutrophils in LN biopsies was significantly higher in LN biopsies compared with CC (Figure 8). It is well-known that HO-1 participates in inflammatory processes, increasing its expression as a regulatory mechanism. However, infiltrating cells in LN biopsies showed low levels of HO-1, as well as circulating monocytes and activated neutrophils, which could be fostering tissue damage and disease development.

## Discussion

Monocytes, macrophages, and neutrophils play an important role in human and murine LN (3, 31, 32). We found that innate immune cells of patients with LN show lower expression of HO-1, have impaired functions at baseline, such as increased phagocytosis of opsonized beads and increased ROS production. ROS level and phagocytosis are reduced when HO-1 expression is induced with CoPP. The predominance of CD14^lo^CD16^hi^ subsets of monocytes could be contributing to the pro-inflammatory environment of LN (33). CD16 receptors expressed in monocytes exhibit high-affinity binding to the FC portion of polymeric IgG antibodies and immune complexes (IC). This interaction leads to activation of monocytes/macrophages and triggers several effector functions, such as phagocytosis, antibody-dependent cell-mediated cytotoxicity, release of inflammatory mediators, and clearance of IC (18–20, 33). In fact, it has been shown that CD16^+^ monocytes and activated neutrophils can exert pro-inflammatory functions secreting TNF-α and interferon (IFN)-γ (32–35), which have been linked to the pathogenesis of LN (32, 36). Serum levels and renal production of these cytokines are increased at the onset of renal disease (32, 34–37), and it has been described in murine models that the use of blocking antibodies against IFN-γ causes a significant delay in disease, stabilizing glomerulonephritis, and significantly decreasing proteinuria, which is associated with improvement in survival (36, 37).

Interestingly, HO-1 has an anti-inflammatory role in immune cells with inhibition of pro-inflammatory signals and recovery of cell homeostasis (38). Various studies have shown that inflamed tissues upregulate the expression of HO-1, which has been proved in gastric ulcers, inflammatory bowel disease, colitis, enteritis, and other diseases (39). The data presented here support that the low expression of HO-1 in monocytes and potentially neutrophils might play an active and relevant role in the pathogenesis of LN, similar to what was observed in multiple sclerosis (MS) (40).

**Figure 9:**
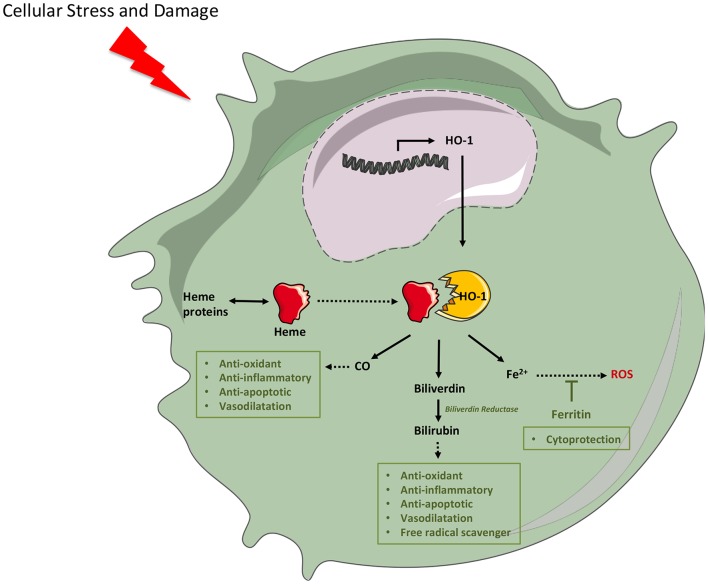
Cytoprotective mechanism of heme oxygenase (HO)-1. Cellular stress and damage release heme that acts as pro-oxidant. Cell injury also induces HO-1 expression through pro-inflammatory signals. HO-1 catabolizes the degradation of heme into free iron (Fe2^+^), carbon monoxide (CO), and bile pigments (biliverdin), which are converted to bilirubin by the enzymatic action of biliverdin reductase. Bilirubin and CO act as potent antioxidants through NADPH oxidase inhibition and reactive oxygen species (ROS) sequestration. Furthermore, they act as anti-inflammatory, antiapoptotic, and produce vasodilatation. Free iron, a pro-oxidant element, is captured by ferritin, which aids in the cytoprotective mechanisms by reducing ROS generation. (This figure was created using Servier Medical Art templates, which are licensed under a Creative Commons Attribution 3.0 Unported License; https://smart.servier.com).

The mechanism responsible for the anti-inflammatory properties of HO-1 is not fully known. However, extensive evidences have demonstrated that the beneficial effects of HO-1 is mediated *via* the breakdown of heme, a vigorous pro-oxidant molecule and a noxious stimulus that amplifies oxidative insult in several models of injury, and the generation of protective products as carbon monoxide (CO), biliverdin with the subsequent formation of bilirubin, and ferritin *via* iron release from the heme moiety (41, 42) (Figure 9). On the other hand, has been described that monocyte HO-1 inhibits protease, nitric oxide and pro-inflammatory cytokine production, immune cell recruitment and infiltration (8, 43); induces anti-inflammatory interferon-beta; and inhibits cell chemoattraction (40, 44) by downregulation of chemokine receptor (45, 46). Furthermore, HO-1 may be an important effector of Foxp3-mediated immune suppression (8).

Disturbances in the phagocytosis of apoptotic cells have been proposed to play a role in the development of autoimmunity, especially in SLE (47–50). Previous works have described defects in the clearance of apoptotic cells in lupus mice and patients (51, 52). In contrast to these reports, our data show an increment in the phagocytosis of monocytes of LN patients. This difference could be explained because most of the published data have evaluated phagocytosis of macrophages that have been *in vitro* differentiated and our work has been focused on monocytes (51, 53, 54). Furthermore, phagocytosis is an extremely complex process, and no single model can account for the diverse structures and outcomes associated with the ingestion of particles. Nevertheless, independently of the type of ingested cell and the receptors involved, several common stages of the process have been established: recognition, engulfment, phagosome maturation (phagolysosome), and degradation (55). The pathways involved in the uptake of apoptotic cells by either macrophages or non-professional phagocytes seem to be unique and very highly conserved in evolution (efferocytosis) (56, 57). However, there are very few data about the underlying mechanisms that drive apoptotic cell phagosome formation and its consequences for subsequent apoptotic cell digestion. In this study, we evaluated the first step of phagocytosis (engulfed material) and in a short period of time (60 min). Based on our results, LN monocytes do not have problems in this initial step of phagocytosis, but it seems that the whole process works until it reaches a critical amount of engulfed beads, as shown by the increase in monocytes that phagocyte four or more beads in LN patients. The accumulation of engulfed material inside monocytes could lead or be explained by a delay or a defect in the subsequent steps of phagocytosis, resulting in poor clearance of apoptotic cells, but more experiments are needed to confirm this statement. The modulation of the phagocytic capacity represents a potential therapeutic target in the control of disease, since apoptotic cells, when not efficiently cleared, may represent a source of autoantigens that drive the autoimmune response in SLE (47–50). Interestingly, here, we demonstrated that the induction of HO-1 modified the phagocytic activity of LN monocytes to a point that they behave similar to HC monocytes. We can therefore speculate that defects in the clearance of apoptotic cells by monocytes of LN patients could be in part explained by reduced levels of HO-1, which could contribute to the initiation and maintenance of autoimmunity. Future studies designed to investigate the phagocytic pathway would be of interest.

In addition, several studies have shown that high levels of ROS have a role in cellular death in SLE; in fact, elevated ROS production has been associated with increased apoptosis, NETosis, and delayed clearance of apoptotic cells (58, 59). Rokutan et al. (58) showed that there are increased oxygen intermediates in the organs of MRL/lpr mice, whereas reduced serum levels of antioxidants and radical scavengers have been reported in human SLE (60). Indeed, treatment with antioxidants, such as vitamin E, suppressed circulating anti-dsDNA antibody levels and the development of renal disease in MRL/lpr mice (61, 62). Different groups have previously shown increased ROS production by LN neutrophils and monocytes. However, there is some controversy in the literature about their response to oxidative stimuli (63, 64), arguing that differences in methodology and patient selection may explain some of these. Here, we observed that LN monocytes have elevated ROS levels at baseline, but HO-1 induction reduces these levels to those of HC, exerting their cytoprotective function (65).

Other groups have demonstrated that HO-1 is upregulated in proximal tubular cells in response to oxidative stress and exerts cytoprotective and anti-inflammatory effects in severe cases of sublethal cellular injury affecting tubular segments, which includes ischemia, nephrotoxins, cytokines, endotoxin, oxidants, and vasoactive substances (66–70). To the best of our knowledge, our group and Kishimoto et al. (12) first examined the expression of HO-1 *in situ* in human LN, focusing on different cell types. Our findings show that HO-1 expression is increased in tubular epithelium compared with infiltrating cells and is decreased in the epithelium when compared to that in HC, but no significant differences were observed between the biopsies of patients with LN and CC biopsies of patients with kidney cancer probably because although the examined tissue was not infiltrated by tumor, it was stressed tissue exposed to an antitumor immune response. Strikingly, although we studied only three HC biopsies, our findings are consistent with what is seen in peripheral monocytes, which show a decreased expression of HO-1 in LN patients. Further studies to confirm the results observed in human biopsies are needed. While LN has been always classified as a glomerular disease, an increasing body of evidence suggests that tubulointerstitial injury might have a prominent role (66). Our results may suggest that HO-1 presence in RTEC could be an attempt of the kidney to protect itself from damage, which is perpetrated, among others, by innate immune cells showing a low expression of HO-1. Since LN patients might express lower levels of HO-1 than HC, they could be having less protective mechanisms against the damage inflicted by inflammatory cells. Furthermore, the induction of HO-1 in the tubular epithelium in proteinuric states that occurs in cases of LN cannot be simply ascribed to increased trafficking of albumin *per se* across the proximal tubule; such expression more likely reflects concomitant injury to tubular epithelial cells occurring *pari passu* with glomerular disease and/or the pro-inflammatory, pro-oxidant, or other perturbing effects of specific proteins or other species appearing in the urinary space, as it has been demonstrated by some authors (67).

In conclusions, we showed that circulating monocytes and activated neutrophils of LN patients have lower levels of HO-1 than those of HC. Although further experiments to demonstrate that these peripheral cells perform tissue damage in LN are needed, our results suggest that this could be the case. In addition, LN monocytes show increased levels of ROS at baseline, and HO-1 induction is able to return HO-1 levels to normal. Thus, decreased HO-1 expression in circulating and infiltrating monocytes and neutrophils of LN patients might promote a pro-inflammatory environment contributing to renal injury. Moreover, we propose that HO-1 induction might exert a cytoprotective role in renal tissue similar to observed in other models of renal injury (68–70) and might regulate innate immunity in LN.

## Data Availability Statement

The raw data supporting the conclusions of this article will be made available upon reasonable request to the corresponding author.

## Ethics Statement

The studies involving human participants were reviewed and approved by Comité Ético Científico Facultad de Medicina Pontificia Universidad Católica de Chile. The patients/participants provided their written informed consent to participate in this study.

## Author Contributions

LC made substantial contributions to the conception and design of the study and drafted the manuscript, performed experimental procedures, made substantial contributions to the analysis and interpretation of data, reviewed the manuscript critically for important intellectual content, and gave final approval of the version to be published. JO participated in the conception and design of the study, performed the qRT-PCR analysis, participated in the analysis and interpretation of data, and revised the final version of the manuscript. PG-M performed immunofluorescence microscopy and histochemical protocol, made substantial contributions to the analysis and interpretation of data, drafted the manuscript and reviewed the manuscript critically for important intellectual content. AV participated in the conception and design of the study, made substantial contributions to the analysis and interpretation of data and reviewed the manuscript critically for important intellectual content. NC participated in the conception and design of the study, performed phagocytosis experiment, participated in analysis of data, drafted the manuscript and reviewed the manuscript critically for important intellectual content. IS participated in the conception and design of the study, performed part of the renal biopsies, participated in the analysis of data, reviewed the manuscript critically for important intellectual content and revised the final version of the manuscript. RV performed experiments, participated in the analysis and interpretation of data, drafted the manuscript, reviewed the manuscript critically for important intellectual content, and revised the final manuscript. GM participated in the conception and design of the study, made substantial contributions to the analysis and interpretation of data, drafted the manuscript and reviewed the manuscript critically for important intellectual content. CL participated in the conception and design of the study, made substantial contributions to the analysis and interpretation of data, and drafted the manuscript. All authors read and approved the final version of the manuscript.

### Conflict of Interest

The authors declare that the research was conducted in the absence of any commercial or financial relationships that could be construed as a potential conflict of interest.
